# The Effect of Honokiol on Ergosterol Biosynthesis and Vacuole Function in *Candida albicans*

**DOI:** 10.4014/jmb.2008.08019

**Published:** 2020-11-27

**Authors:** Lingmei Sun, Kai Liao

**Affiliations:** 1 Department of Pharmacology, Medical School of Southeast University, Nanjing 20009, P.R. China; 2 Department of Pathology and Pathophysiology, Medical School of Southeast University, Nanjing 10009, P.R. China

**Keywords:** *Candida albicans*, honokiol, ergosterol biosynthesis, vacuole

## Abstract

Ergosterol, an essential constituent of membrane lipids of yeast, is distributed in both the cell membrane and intracellular endomembrane components such as vacuoles. Honokiol, a major polyphenol isolated from *Magnolia officinalis*, has been shown to inhibit the growth of *Candida albicans*. Here, we assessed the effect of honokiol on ergosterol biosynthesis and vacuole function in *C. albicans*. Honokiol could decrease the ergosterol content and upregulate the expression of genes related with the ergosterol biosynthesis pathway. The exogenous supply of ergosterol attenuated the toxicity of honokiol against *C. albicans*. Honokiol treatment could induce cytosolic acidification by blocking the activity of the plasma membrane Pma1p H^+^-ATPase. Furthermore, honokiol caused abnormalities in vacuole morphology and function. Concomitant ergosterol feeding to some extent restored the vacuolar morphology and the function of acidification in cells treated by honokiol. Honokiol also disrupted the intracellular calcium homeostasis. Amiodarone attenuated the antifungal effects of honokiol against *C. albicans*, probably due to the activation of the calcineurin signaling pathway which is involved in honokiol tolerance. In conclusion, this study demonstrated that honokiol could inhibit ergosterol biosynthesis and decrease Pma 1p H^+^ -ATPase activity, which resulted in the abnormal pH in vacuole and cytosol.

## Introduction

*Candida albicans*, an opportunistic fungal pathogen in humans, is a common microbe residing on the oral mucosa, skin, and intestinal tract in healthy individuals [1, 2]. It is worth noting that it may cause life-threatening invasive infections in immunocompromised patients and is listed as the fourth most common pathogenic microorganism of nosocomial bloodstream infections [2]. Several antifungal agents act by inhibiting the biosynthesis of ergosterol [3, 4]. Both the reduced ergosterol and the excess of intermediate sterols are able to induce plasma membrane disorder and eventually lead to cell growth inhibition [5, 6]. Ergosterol, similar to cholesterol in mammals, is an essential constituent of membrane lipids. It modulates the fluidity, permeability, integrity, and thickness of the cell membrane [6]. A significant amount of ergosterol is found both in the cell membrane and in intracellular endomembrane components such as vacuoles [7, 8]. Pma1p, which encodes the plasma membrane H^+^-ATPase, is the primary moderator of cytosolic pH in fungi. Its function is to extrude cytoplasmic hydrogen ion to maintain neutral-to-alkaline pH in the cytosol, and therefore, it is a potential drug target due to its necessity and specificity to fungi [7, 8].

The vacuole, analogous to the mammalian lysosome and the largest organelle in fungal cells, plays an essential role in a variety of cellular functions including cellular response to environmental stresses, ionic homeostasis, and the yeast-hyphae transition [9-11]. Vacuoles are needed to maintain intra-vacuolar pH and ion homeostasis, which is modulated by the vacuolar proton-translocating ATPase (V-ATPase) [12]. Cells show an abnormal morphology of the vacuole and a reduced degree of vacuolar acidification after treatment with ergosterol biosynthesis inhibitors such as azoles or morpholines [8]. V-ATPase is found in all eukaryotic cells and is responsible for the acidification of intracellular compartments by driving the translocation of protons across the vacuolar membrane into the lumen [8, 12]. Therefore, V-ATPase is critical for maintaining cellular ion homeostasis.

*Magnolia officinalis*, a plant with a long history of use in traditional Asian medicine, is administered clinically to treat bacterial infections, inflammation, and gastrointestinal diseases [13-15]. Honokiol, a bioactive polyphenol, is one of the main active ingredients in *M. officinalis*. Honokiol has been found to induce reactive oxygen species accumulation through mitochondrial dysfunction in *C. albicans* [16, 17]. Recently, it has been shown that altered sterol metabolism could decrease Fe-S cluster synthesis, and thus decrease mitochondrial function [18]. To further reveal the underlying mechanism of honokiol against *C. albicans*, we studied the effect of honokiol on ergosterol biosynthesis and vacuole function in *C. albicans*.

## Materials and Methods

### Materials

Honokiol (5,5’-diallyl-2,4’-dihydroxybiphenyl) was obtained from Xi'an Yuquan Biological Technology Co., Ltd. and its purity was over 98% as analyzed by high-performance liquid chromatography. Yeast vacuole membrane marker MDY-64 was purchased from Invitrogen (Thermo Fisher Scientific, USA). EGTA, ergosterol, and amiodarone (AMD) were purchased from Aladdin Bio-Chem Technology Co., Ltd. (China). For analysis of exogenous sterol utilization, ergosterol was dissolved in a mixture of ethanol (50%) and Tween-80 (50%) to give a 10mM stock solution, which was used to supplement the liquid medium with a final concentration of 50 μM. The same concentration of vehicle without ergosterol was used as a control.

### Strains and Media

*C. albicans* strain SC5314 was stored with 15% glycerol as frozen stock at –80°C. Before each experiment, cells were freshly revived on YPD (yeast extract/ peptone/dextrose) agar plate from the stock.

### Ergosterol Quantification

An overnight liquid culture of *C. albicans* SC5314 was diluted 100-fold with YPD medium and exposed to drug treatment. After 12 h incubation, the cells were harvested by centrifugation. Cell total sterol was extracted and measured as previously described [19].

### Quantitative RT (qRT)-PCR Assay

C. albicnas strain SC5314 was grown overnight in YPD medium. Yeasts cells which were resuspended at a cell density of 1.0 × 10^7^ cells/ml were treated with 16 μg/ml honokiol for 12 h at 30°C. Total RNA was extracted by the hot phenol method [19]. About 1 μg of RNA was reverse-transcripted into cDNA using AMV reverse transcriptase (Promega, USA). Primer sequences of genes are shown in supplementary Table S1. The qRT-PCR and data analysis were conducted as previously described [16, 17].

### Measurement of Acidification of the External Medium

The P-type H^+^-ATPase has been proved to relate with ergosterol-enriched domains [7, 8]. It pumps protons out of cells to acidify the external environment as soon as glucose is activated [7, 8]. To estimate the effect of honokiol on the function of Pma1, we tested acidification of the extracellular medium via glucose activation in honokiol-treated yeast cells. The effect of honokiol on glucose-induced acidification of the external medium was tested according to the method as previously described [20].

### Isolation of Plasma Membrane and Measurement of ATP Hydrolysis

Isolation of plasma membrane was performed as described previously [20]. The ATP hydrolysis method was tested in a medium (0.2 mM EDTA, 60 mM Tris, 8 mM MgCl_2_, pH 5.7). After incubating the plasma membrane at 37°C for 10 min with or without honokiol, the reaction was triggered by the addition of ATP (50 mM) and then incubated for 10 min. Cold trichloroacetic acid (5%) was added to stop the progress of the reaction. Free inorganic phosphate was analyzed as described previously [20].

### Vacuole Staining and Vacuolar pH Measurements

MDY-64, a yeast vacuole membrane marker, was used to visualize vacuoles according to the manual description. Vacuole morphology was monitored by fluorescence microscopy using an FITC filter set (Olympus IX71, Olympus Co., Japan). Quinacrine, which shows green fluorescence in acidic condition, was employed to estimate the vacuolar pH in living *C. albicans* cells qualitatively [21]. Yeast cells were exposed to drugs for 4 h, and then the cells were stained with 200 μM quinacrine. The fluorescence images of cells were obtained by the fluorescence microscope using the FITC filter set.

### Measurement of the Relative Levels of Intracellular Free Ca^2+^

The relative levels of intracellular free Ca^2+^ were determined by the Ca^2+^-sensitive indicator Fura 2-AM. *C. albicans* strain was cultured overnight at 30°C in YPD medium and washed with PBS buffer, and 1 × 10^7^ cells were resuspended in PBS (without CaCl_2_ and MgCl_2_) plus 10 μM Fura 2-AM at 37°C for 1 h. After washing three times with PBS, Fura-2 fluorescence was determined by excitation wavelength at 340 nm and 380 nm, and emission wavelength at 510 nm in a BioTek Synergy 4 microplate reader (BioTek Instruments, Inc.), with relative levels of intracellular Ca^2+^ being expressed as the ratio of the fluorescence intensity upon excitation at 340 nm (F_340_) to the F_380_.

## Results

### Honokiol Inhibits Ergosterol Biosynthesis

**Fig. 1:**
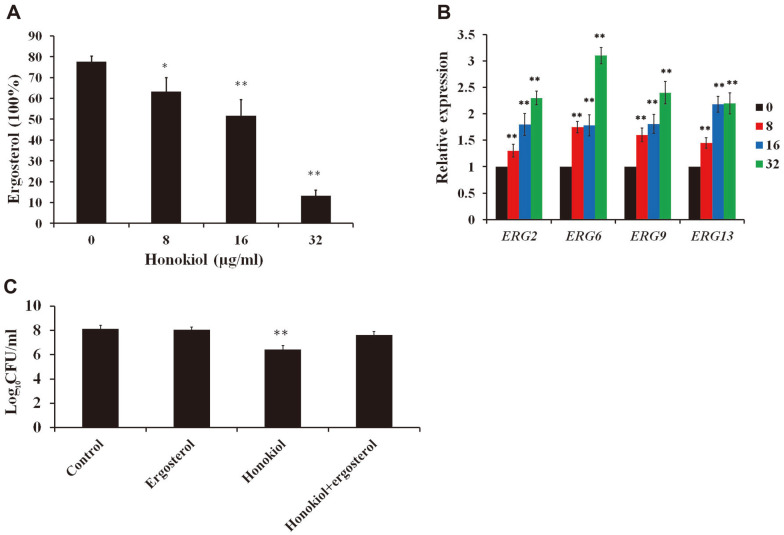
The effect of honokiol on ergosterol biosynthesis. (**A**) Honokiol decreased the ergosterol level in *C. albicans*. Ergosterol was extracted from cells treated by different concentrations of honokiol and measured by the spectrophotometric method. (**B**) qRT-PCR of the genes involved in ergosterol biosynthesis. *C. albicans* was treated with different concentrations of honokiol for 12 h. (**C**) The effect of exogenous ergosterol on the antifungal activity of honokiol. *C. albicans* was treated with honokiol (16 μg/ml), ergosterol (50 μM) or their combination. *p < 0.05; **p < 0.01.

Ergosterol is an essential component of yeast cell membrane lipids [6]. Because of its absence in mammals, targeting ergosterol is appealing and effective as a therapeutic avenue [3, 5]. To verify if honokiol could inhibit ergosterol biosynthesis, we measured the cellular ergosterol level using a spectrophotometric assay. As expected, the ergosterol content significantly decreased in the cells treated with honokiol compared with the control group (Fig. 1A). To further investigate the effect of honokiol on the expression of ergosterol biosynthesis genes, real-time RT-PCR analysis was employed. The results showed the upregulation of ergosterol synthesis genes in response to honokiol treatment (Fig. 1B). Also, feeding exogenous ergosterol could reverse the inhibition of cell growth caused by honokiol treatment (Fig. 1C). The ability of exogenous ergosterol to restore the cell growth after honokiol treatment supports a hypothesis that antifungal activity of honokiol is partly a result of the ergosterol depletion.

### Honokiol Disrupts H^+^-ATPase Activity

**Fig. 2:**
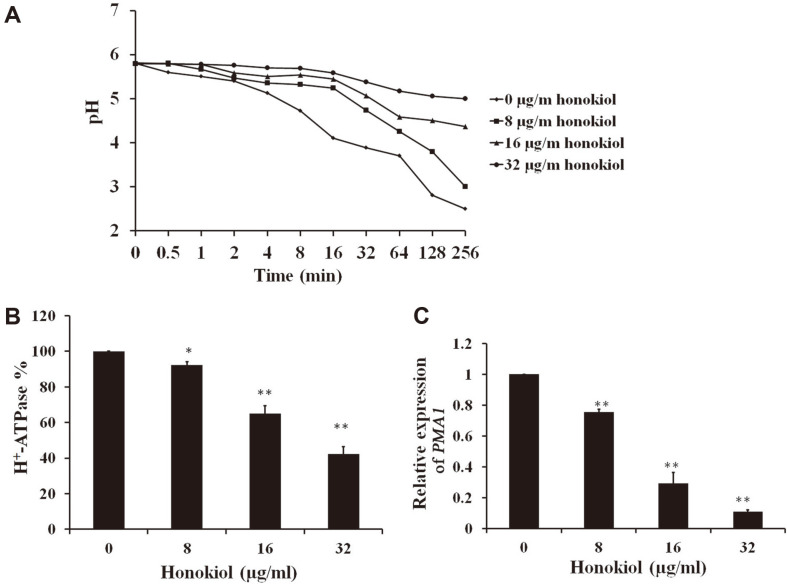
Honokiol impaired the function of Pma1p. (**A**) The inhibitory effect of honokiol on the glucose-dependent acidification of medium in *C. albicans*. Cells were diluted to 3~5 × 10^7^ CFU/ml. Extracellular pH was recorded after glucose was added to 2% at time 0. A representative of several experiments with very similar results is shown. (**B**) Honokiol decreased plasma membrane ATPase activity. The percentage of ATPase-specific activity in isolated plasma membranes treatment by different concentrations of honokiol as compared to ATPase-specific activity in the control was calculated. (**C**) The effect of honokiol on the expression of the *PMA1* gene. *C. albicans* was treated with different concentrations of honokiol for 12 h. Bars represent mean ± SD. *p < 0.05; **p < 0.01.

The plasma membrane H^+^-ATPase Pma1, the primary modulator of cytosolic pH in fungi, has been demonstrated to associate with ergosterol-enriched domains [7, 9]. To estimate the effect of honokiol on Pma1 function, we measured the acidification of the extracellular medium upon glucose activation in honokiol treatment cells. As shown in Fig. 2A, the acidification curve of the extracellular medium was revealed to be quite different between the control and honokiol-treated group. Honokiol treatment slowed the medium acidification rate relative to wild type, as shown in Fig. 2A. Differences between control and honokiol-treated group in acidification of their external environments indicate a significantly reduced H^+^-ATPase activity in the strain with honokiol treatment compared to the control group. We isolated the plasma membranes and detected the ATPase-specific activity. Treatment by honokiol led to decrease in H^+^-ATPase activity in the plasma membrane in a concentration-dependent manner (Fig. 2B). We further measured the expression of the *PMA1* gene. As expected, honokiol treatment resulted in a lower level of *PMA1* gene expression than in the control group (Fig. 2C). This low level of *PMA1* gene expression could also result in a low level of Pma1p. Together these findings suggest that honokiol could decrease the amount of Pma1p in the plasma membrane as well as the activity of the Pma1p enzyme itself.

### Honokiol Affects Vacuole Morphology and Acidification

**Fig. 3:**
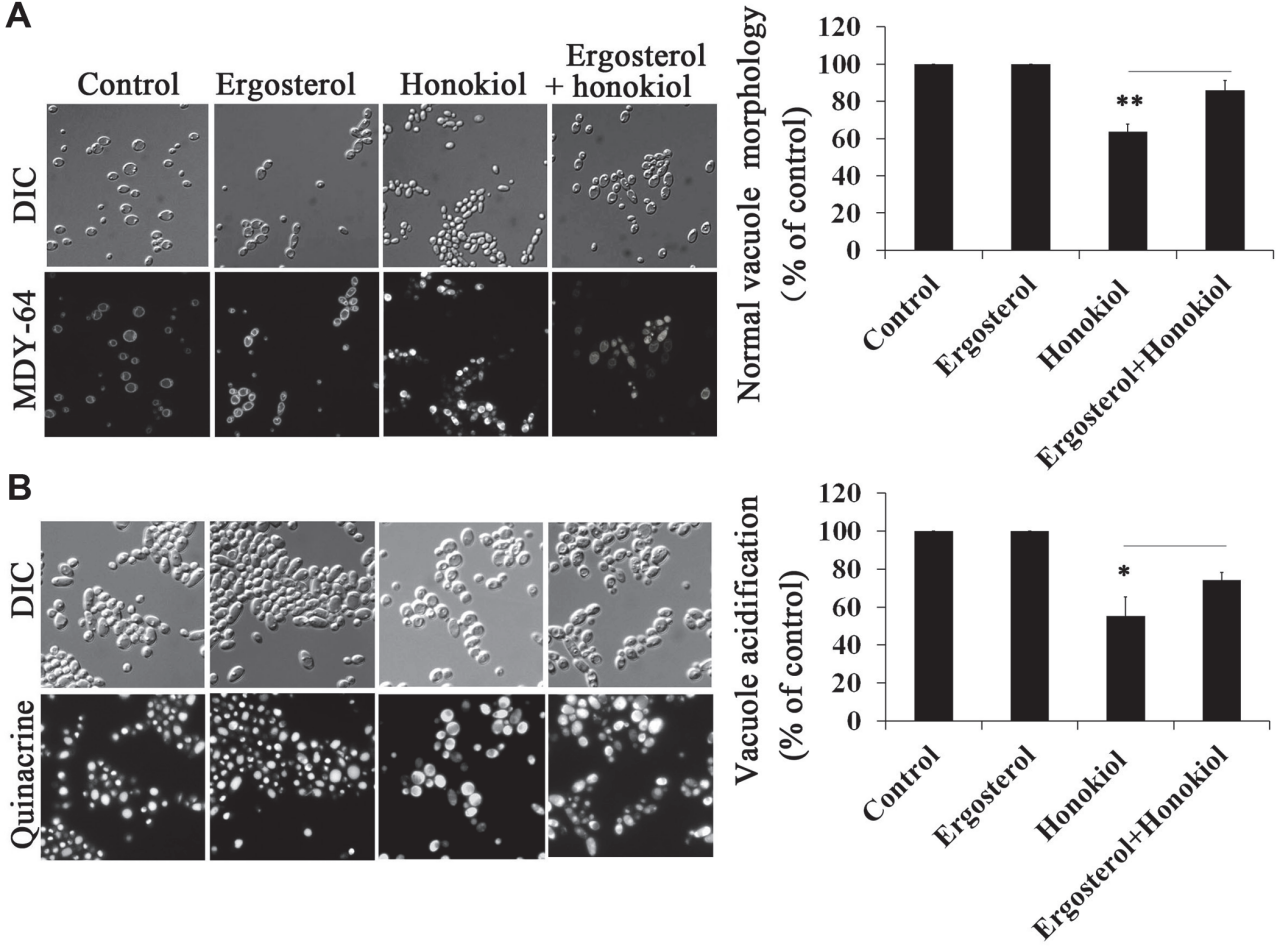
The effect of honokiol on the vacuole morphology and acidification in *C. albicans*. (**A**) Vacuole morphology was observed using the fluorescein stain yeast vacuole marker MDY-64. (**B**) Vacuole acidification was assessed by quinacrine which is accumulated in the acidic compartment. *C. albicans* was treated with honokiol (16 μg/ml), ergosterol (50 μM) or their combination. Bars represent mean ± SD. *p < 0.05; **p < 0.01.

To investigate the effect of honokiol on the vacuole, we analyzed the vacuole structure and function after treatment by honokiol. We used the yeast vacuole marker MDY-64 to stain yeast vacuolar membranes. As shown in Fig. 3A, the vehicle control strains exhibited the typical ring-staining pattern of the vacuole membrane. In contrast, yeast vacuole marker MDY-64 diffusely distributed in the cytoplasm in the honokiol-treated strains. This result suggested honokiol induced abnormal vacuole morphology. We also assessed the effect of the exogenous supply of ergosterol on vacuole morphology treatment by honokiol. As shown in Fig. 3A, compared with cells treated with honokiol alone, ergosterol decreased the abnormal vacuole morphology to some degree.

A variety of methods can be used to assess the relative acidification of the vacuole [22]. Quinacrine, a weak base, is frequently used to label the vacuole [21]. Because of its lipophilic nature, quinacrine is able to diffuse across the vacuolar membrane. Still, once exposed to the low pH of the vacuolar lumen it becomes protonated and is unable to leave the organelle [22]. In the vehicle control group, the vacuole was clearly labeled with quinacrine. However, under honokiol treatment, quinacrine fluorescence did not colocalize within the vacuole lumen, but was diffusely distributed in the cytoplasm (Fig. 3B), and therefore appears to be defective in vacuolar acidification. The impaired trafficking of quinacrine to the vacuolar lumen in the honokiol-treated strains may be caused by cytosolic acidification and vacuolar alkalization. Moreover, the exogenous ergosterol could recover the localization of quinacrine to some extent (Fig. 3B).

### Honokiol Disrupts Intracellular Calcium Homeostasis

**Fig. 4:**
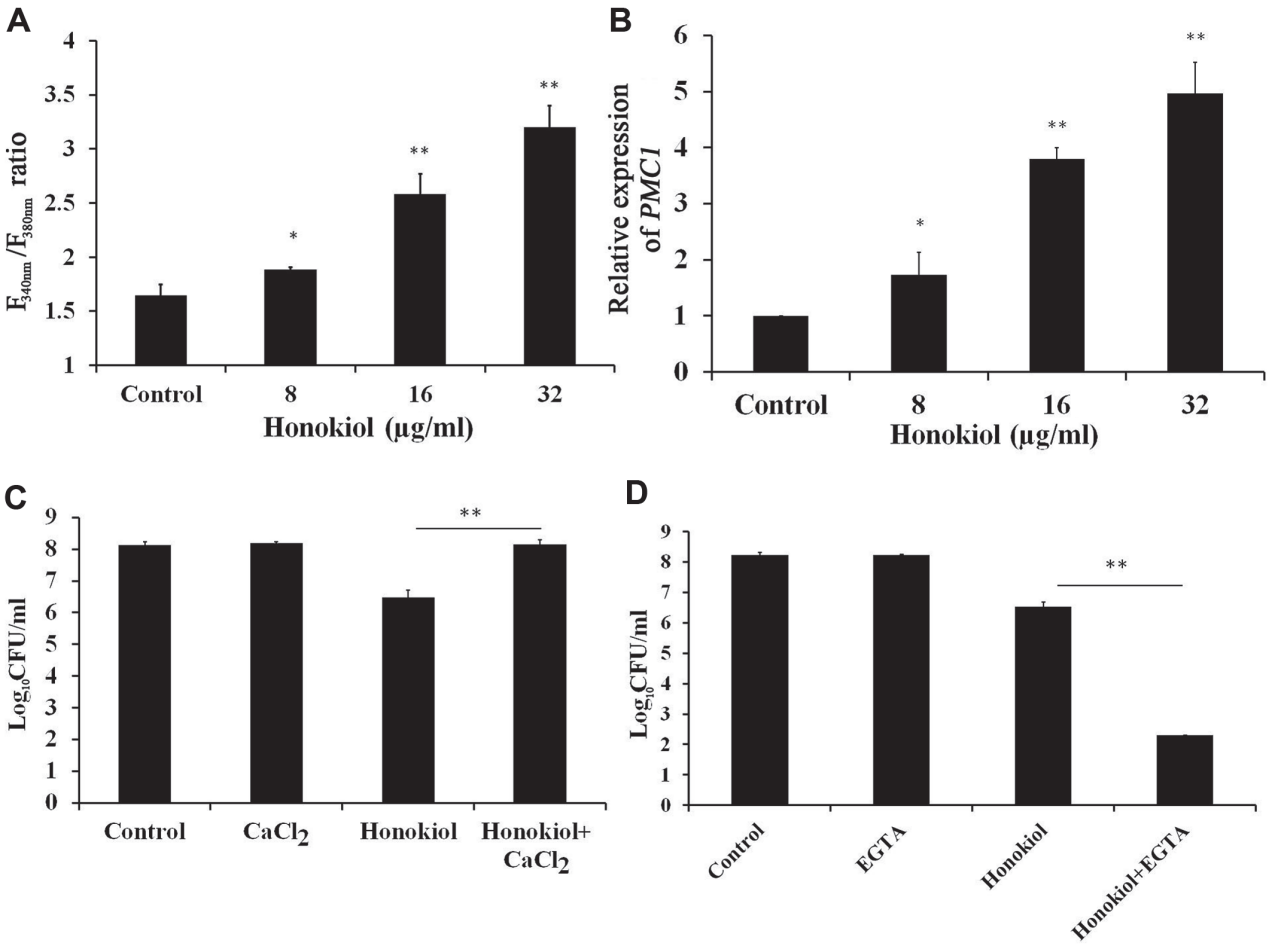
The intracellular calcium level was related with honokiol tolerance against *C. albicans*. (**A**) Honokiol treatment induced abnormal intracellular calcium levels. Cells were grown in YPD liquid medium at 30°C, labeled with calcium-responsive dye Fura 2-AM. The fluorescence intensity was tested with excitation at 340 nm or 380 nm and emission at 510 nm. Intracellular calcium levels are expressed as F_340_/F_380_ ratios. (**B**) The effect of honokiol on the expression of *PMC1* gene. *C. albicans* was treated with different concentrations of honokiol for 12 h. (**C** and **D**) The effect of exogenous calcium (**C**) or calcium chelator EGTA (**D**) on the antifungal activity of honokiol. *C. albicans* was treated with honokiol (16 μg/ml), calcium (10 mM) / EGTA (10 mM) or their combination. Bars represent mean ± SD. *p < 0.05; **p < 0.01.

In the fungal cell, more than 90% of the intracellular Ca^2+^ is stored in the vacuole. Maintenance of calcium homeostasis is essential for yeast cells to resist environmental stresses such as drug exposure. Hence, to observe whether the vacuolar trafficking defects by honokiol impact cellular Ca^2+^ homeostasis, the calcium-sensitive dye Fura 2-AM was employed to detect the relative levels of intracellular free calcium. As shown in Fig. 4A, the F_340_/F_380_ ratio of Fura 2-AM-stained honokiol-treated cells was significantly higher than that of the vehicle-treated group, suggesting that honokiol-treated cells have abnormally high intracellular Ca^2+^ levels (Fig. 4A). Pmc1, the yeast Ca^2+^ pump localizes to the vacuolar membrane Ca^2+^-ATPase, is induced under calcium stress and serves to detoxify excess Ca^2+^ by sequestration into the vacuole [23]. Fluconazole treatment could strongly induce the expression of *PMC1* gene, a downstream effector of calcineurin signaling [23, 24]. In our study, *PMC1* expression was also enhanced approximately fourfold in cells exposed to honokiol (16 μg/ml) compared with control cells (Fig. 4B). The possibility was raised that *PMC1* could play a survival role in the presence of honokiol. Also, the exogenous calcium addition in the medium which could activate calcineurin enhanced the tolerance to honokiol (Fig. 4C). When we used the Ca^2+^ chelator EGTA to bind free Ca^2+^ in the medium, the supplement of 10 mM EGTA increased the susceptibility of honokiol against *C. albicans* (Fig. 4D). Taken together, our results support the increase of honokiol-induced intracellular Ca^2+^ levels as a protective effect against honokiol-induced cell death.

### In Vitro Interactions between Honokiol and AMD

**Fig. 5:**
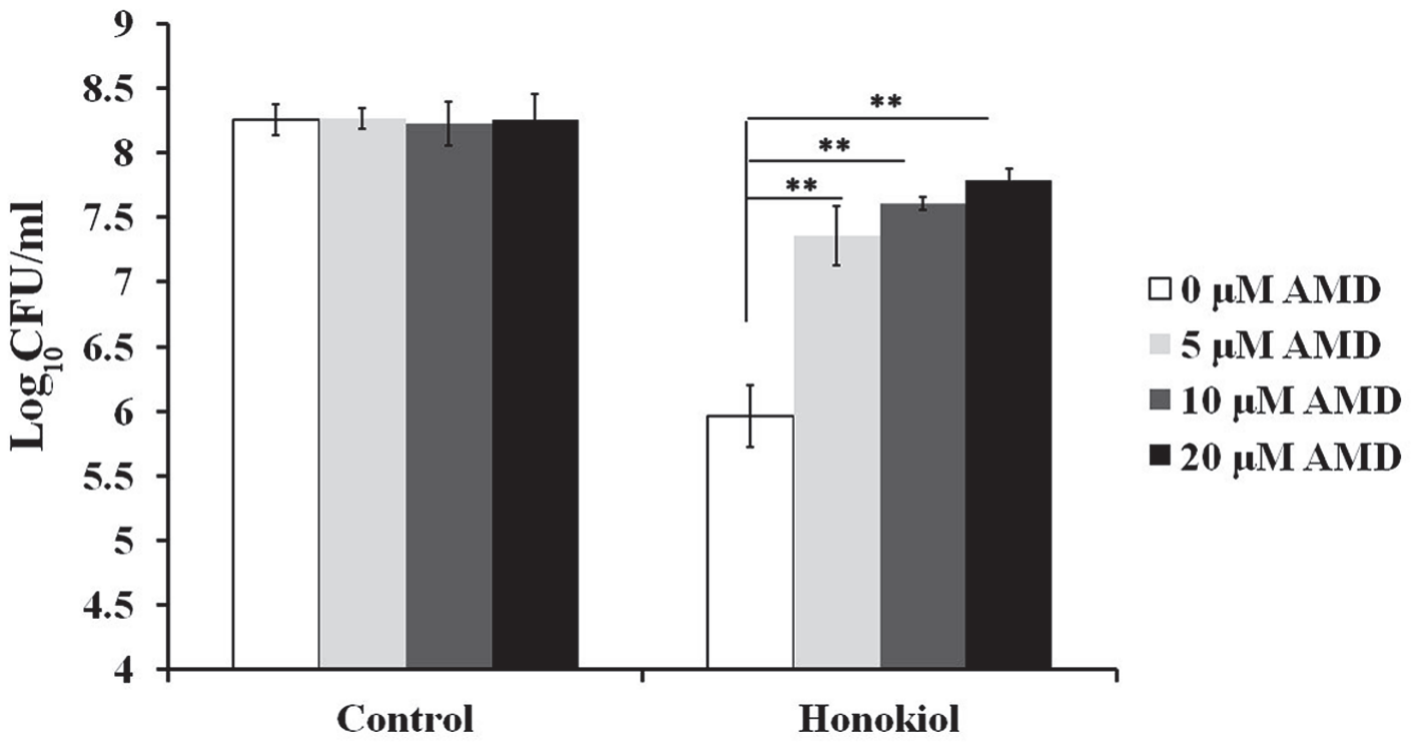
The effect of AMD on the antifungal activity of honokiol. *C. albicans* was treated with honokiol (16 μg/ml), AMD (5, 10, 20 μM) or their combination. Bars represent mean ± SD. **p < 0.01.

AMD, an antiarrhythmic drug, has a prominent and rapid effect on *C. albicans*, which is most noticeably reflected as changes in calcium stress pathways [25]. It could elicit an immediate influx of calcium and the increased the expression of calcineurin-regulated C2H2 transcription factor *CRZ1* [8, 25]. To assess the effect of AMD on the anti-candidal activity of honokiol, we added various amounts of AMD to medium with or without honokiol. As shown in Fig. 5, culture containing AMD and honokiol combinations were found to have a significant increase in CFU relative to culture without AMD. This result suggests that AMD-induced calcium influx could alleviate honokiol toxicity.

## Discussion

As a primary constituent of cell membranes, the depletion of ergosterol alters many intracellular biological reactions and changes membrane properties such as fluidity, permeability, and thickness [3,4]. Ergosterol also has essential roles in mitochondria, vacuoles, and lipid rafts in *C. albicans* [8]. Due to the absence of ergosterol in mammals, ergosterol as a target of antifungal drugs has excellent advantages [8, 26, 27]. In our study, honokiol could decrease the ergosterol content and disturb the expression of genes related to ergosterol biosynthesis (Figs. 1A and 1B). The upregulation of global *ERG* genes was also observed following exposure to ergosterol biosynthesis inhibitors in *C. albicans* [18, 28, 29]. However, the molecular mechanism behind this compensatory response pathway is still mostly unknown. Furthermore, concomitant addition of ergosterol restored the cell growth after honokiol treatment in *C. albicans* (Fig. 1C). Thus, depletion of ergosterol is a plausible mechanism for the antifungal activity of honokiol.

The P-type H^+^-ATPase Pma1 related to ergosterol-enriched domains is the primary regulator of cytosolic pH in fungi [7, 8]. Upon glucose activation, it extrudes protons out of cells to maintain normal intracellular pH and acidifies the extracellular medium [30, 31]. The lack of ergosterol in the plasma membrane, as well as a reduced level of ATP after honokiol treatment as demonstrated previously in *C. albicans* may cause the abnormal functioning of H^+^-ATPase (Fig. 2). We also observed the lower expression levels of the *PMA1* gene in *C. albicans* treatment by honokiol (Fig. 2C). Honokiol blocks Pma1p H^+^-ATPase by inhibiting the transport of cytosolic protons out of the cell. Given all these results, we reasoned that honokiol treatment could induce cytosolic acidification by blocking the activity of Pma1p H^+^-ATPase.

The vacuole, the largest organelle in yeast cells, plays an essential role in multiple intracellular functions such as the response to environmental conditions and the yeast-to-hyphae transition [8-10]. Vacuoles are needed to maintain the intravacuolar homeostasis of pH and ion [32]. In our study, we used two fluorescent dyes, the yeast vacuole membrane marker MDY-64 and the weak base quinacrine, to observe the effect of honokiol on the morphology of the vacuole and vacuolar acidification, respectively (Figs. 3A and 3B). We observed that honokiol induced the abnormal morphology and defective acidification of the vacuole (Fig. 3B). There are previously reported data that cells with *ERG2* or *ERG24* deletion, or exposure to ergosterol biosynthesis inhibitors morpholines or azoles, decrease the degree of vacuolar acidification and show an abnormal morphology of the vacuole [33]. On this account, we infer that honokiol induced the damage to vacuole morphology and function partly due to ergosterol depletion. A previous study suggested a role for sphingolipids in helping to maintain vacuole morphology and function [34]. Myriocin, a metabolite isolated from the insect fungus *Isaria sinclairii*, irreversibly inhibits serine palmitoyltransferase, and thus decreases the sphingolipid synthesis [35]. Both ergosterol and sphingolipid depletion could induce abnormal vacuole morphology and function (Fig. S1). Furthermore, increased levels of cytosolic calcium correlate with vacuolar fragmentation [36]. Further studies are needed to research the mechanism of honokiol on abnormal vacuolar morphology and function.

Maintenance of calcium homeostasis is essential for cells to respond to various stresses [37]. The vacuole is a major calcium store in many organisms, particularly plants and fungi [23]. Upon appropriate stimulation, calcium is released rapidly from extracellular sources as well as intracellular stores into the cytoplasm to activate calcium-dependent protein molecules such as calcineurin [25, 38]. In this study, honokiol treatment induced the accumulation of calcium and the upregulation of *PMC1* (Figs. 4A and 4B). Upon the extra calcium accumulation, Pmc1p localizing to the vacuolar membrane could trap excess calcium into the vacuole. Also, the increase in calcium accumulation could lead to activate the calcineurin signaling pathway [23]. The exogenous calcium weakened the toxicity of honokiol against *C. albicans* (Fig. 4C). By contrast, calcium chelator EGTA increased the susceptibility of honokiol against *C. albicans* (Fig. 4D). This is probably due to the activation of the calcineurin signaling pathway being involved in honokiol tolerance against *C. albicans*.

In *Saccharomyces cerevisiae*, AMD could increase Ca^2+^ influx, so it affects calcium homeostasis, leading to the activation of the calcineurin pathway [8, 25]. A previous study provided in vitro evidence that AMD is obviously synergistic with azoles against *C. albicans* [39]. On the contrary, in our study, AMD attenuated the antifungal activity of honokiol against *C. albicans* (Fig. 5). Due to the AMD and honokiol combination yielding a mean increase of Log_10_CFU/ml less than 2 Log_10_CFU/ml compared with honokiol treatment alone, the interaction of AMD and honokiol was indifferent. This indifferent interaction was also determined using the checkerboard method (data not shown).

**Fig. 6:**
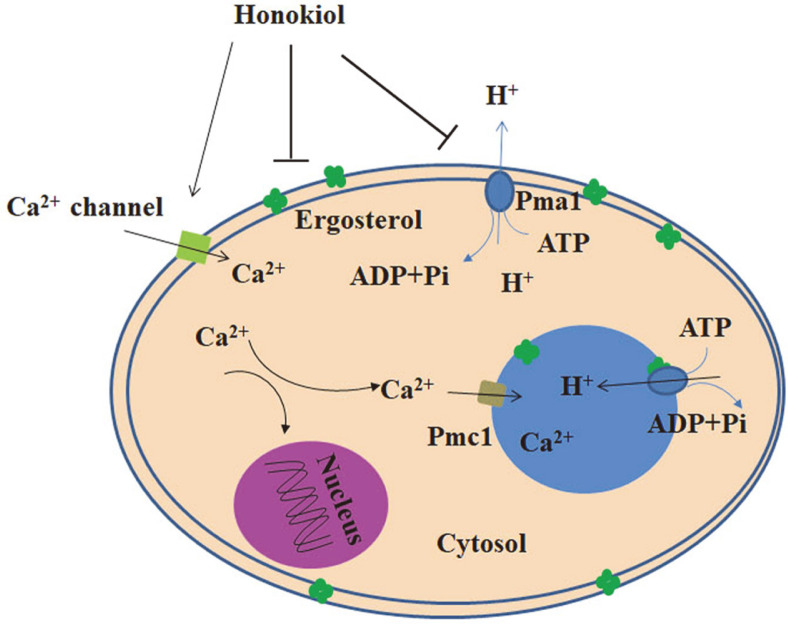
A hypothetical model for the effect of honokiol on ergosterol biosynthesis and vacuole function in *C. albicans*.

Overall, the present study supports evidence that honokiol inhibits ergosterol biosynthesis and decreases Pma1 H^+^-ATPase activity, resulting in abnormal pH in the vacuole and cytosol (Fig. 6). The increased calcium level has been shown to induce adaptive responses that assist *C. albicans* survival following honokiol treatment. The results of this study will contribute to a better understanding of the antifungal mechanism of honokiol.

## Supplemental Materials



Supplementary data for this paper are available on-line only at http://jmb.or.kr.
