# Supplementary material for: The Effect of Honokiol on Ergosterol Biosynthesis and Vacuole Function in Candida albicans
Source: J Microbiol Biotechnol. 2020 Nov 27;30(12):1835–42. doi: 10.4014/jmb.2008.08019 (PMC9728367; doi:10.4014/jmb.2008.08019)
Supplement: Supplementary file 1 [file JMB-30-12-1835-supple.pdf]

J. Microbiol. Biotechnol.
https://doi.org/10.4014/jmb.2008.08019

Table S1. Primers for real-time PCR

Gene	Forward primer (5’-3’)	Reverse primer (5’-3’)	Product length
ERG2	TCCTGGTGCATTGATTCCCG	ATGATTCACCGGGCATAGCA	95
ERG6	AACCAGGTGGTGTTTTCGGT	AGCTTGTTCAGCAACTTTACGA	146
ERG9	TGCATTGGGTCATGTCACTG	GCCACTGCCATTACTTGAGGA	99
EG13	GTTAACGCTTGTTACGGTGGT	GGCAGCACCTTTGTCGTAGA	129
PMA1	AGGGTGCCCCATTATTCGTC	GAAGCAAATTCGGCAACGGT	100
PMC1	AATTGCTTTGGCCCATCGTG	TCCTTTGCAGTGGTTGTGGT	102
ACT1	ACGGTGAAGAAGTTGCTGCT	GGAAAACAGCTCTTGGAGCG	93

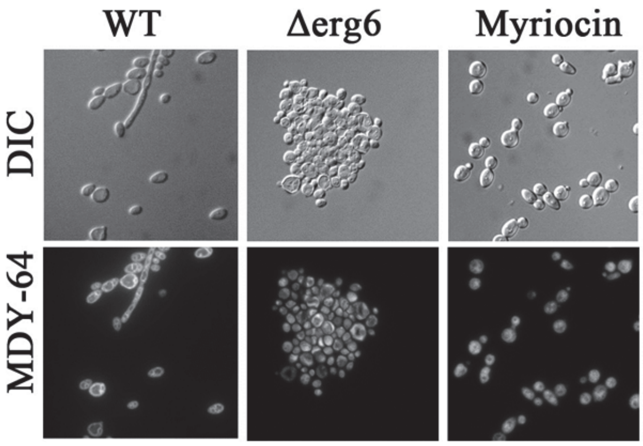

Fig.S1. Vacuole morphology was observed using the fluorescein stain yeast vacuole marker MDY-64.
